# Cross-cultural adaptation and validation of the Spanish version of the Oxford Hip Score in patients with hip osteoarthritis

**DOI:** 10.1186/s12891-017-1568-3

**Published:** 2017-05-22

**Authors:** Jesús Martín-Fernández, Pedro Gray-Laymón, Antonio Molina-Siguero, Javier Martínez-Martín, Roberto García-Maroto, Isidoro García-Sánchez, Lidia García-Pérez, Vanesa Ramos-García, Olga Castro-Casas, Amaia Bilbao

**Affiliations:** 1 C° Villamanta (C.S. Navalcarnero). Gerencia Asistencial de Atención Primaria. Servicio Madrileño de Salud, Madrid, Spain; 2 Facultad de Ciencias de la Salud, Universidad Rey Juan Carlos, Madrid, Spain; 3 Red de Investigación en Servicios Sanitarios y Enfermedades Crónicas (REDISSEC), Madrid, Spain; 4 Servicio de Cirugía Ortopédica y Traumatología, Hospital Universitario Rey Juan Carlos, Madrid, Spain; 5 C.S. Presentación Sabio, Gerencia Asistencial de Atención Primaria, Servicio Madrileño de Salud, Madrid, Spain; 6 Servicio de Cirugía Ortopédica y Traumatología, Hospital Universitario Fundación Alcorcón, Madrid, Madrid Spain; 7 Servicio de Cirugía Ortopédica y Traumatología, Hospital Universitario Clínico San Carlos, Servicio Madrileño de Salud, Madrid, Spain; 8 Servicio de Traumatología y Cirugía Ortopédica, Hospital Galdakao-Usansolo (Osakidetza), Galdakao (Bizkaia), Spain; 9 Fundación Canaria de Investigación Sanitaria (FUNCANIS), Santa Cruz de Tenerife, Spain; 10 Red de Investigación en Servicios Sanitarios y Enfermedades Crónicas (REDISSEC), Santa Cruz de Tenerife, Spain; 11 Unidad de Investigación, Hospital Universitario Basurto (Osakidetza) – Red de Investigación en Servicios de Salud en Enfermedades Crónicas (REDISSEC), Bilbao (Bizkaia), Spain

**Keywords:** Quality of life, Health status, Osteoarthritis, Hip, Validation studies

## Abstract

**Background:**

Osteoarthritis (OA) of the hip is a disease that entails a major burden for patients and the society as a whole. One way of measuring this burden for the patient is through impact on Health-related Quality of Life (HRQL). The Oxford Hip Score (OHS) is a well-known tool to measure HRQL in patients with OA of the hip. This study aims to assess the psychometric properties of the Spanish-adapted version of the OHS, including its reliability, validity, and sensitivity to change.

**Methods:**

Prospective observational study that included 361 patients diagnosed with hip OA (according to the criterion of the American College of Rheumatology) from 3 different Spanish regions. Their HRQL was assessed using a generic questionnaire, the EQ-5D-5 L, and two specific ones (the Western Ontario and McMaster Universities Osteoarthritis Index, WOMAC, and the OHS) adapted to Spanish. There was a follow-up period of 6 months, and the acceptability, psychometric properties, presence of ceiling and floor effects, validity, reliability, and sensitivity to changes of the OHS were measured.

**Results:**

The OHS was fully answered in 99.4% of cases with no indication of ceiling or floor effects. Its factor structure can be explained in a single dimension. Its discriminative capacity was very good compared to the groups generated by the WOMAC and the EQ-5D-5 L. The correlation between the OHS and dimensions of the WOMAC or EQ-5D-5 L utilities was ≥0.7. Excellent test-retest reliability (ICC = 0.992; CI95%: 0.994–0.998) and internal consistency (Cronbach’s α = 0.928) were observed. The minimal clinically important difference (MCID) was 7.0 points, and the minimum detectable change (MDC) was 5.5 points. The effect size for moderate improvement in perceived HRQL was 0.73, similar to that of WOMAC dimensions and higher than the EQ-5D-5 L.

**Conclusions:**

The Spanish-adapted version of the OHS is a useful, acceptable tool for the assessment of perceived HRQL in patients with hip OA, and has psychometric properties similar to those of the WOMAC that allow for discriminating both a patient’s condition at a given moment and changes that can occur over time.

**Electronic supplementary material:**

The online version of this article (doi:10.1186/s12891-017-1568-3) contains supplementary material, which is available to authorized users.

## Background

Osteoarthritis (OA) is the most frequent joint disease, manifesting when structural changes in the joint cause pain and functional impairment. The prevalence of hip OA is high and is augmenting in developed countries due to increases in life expectancy and obesity pandemics [1, 2]. In a literature review by Pereira et al., the prevalence of hip OA was reported to be 10.9% (CI 95%: 10.6–11.2), although the figure was higher when based on radiological diagnosis rather that clinical evidence [3]. The prevalence of hip OA in Spain has been estimated to be 0.9% in the population >40 years of age [4], and 7.4% for people >60 years of age [5].

**Fig. 1:**
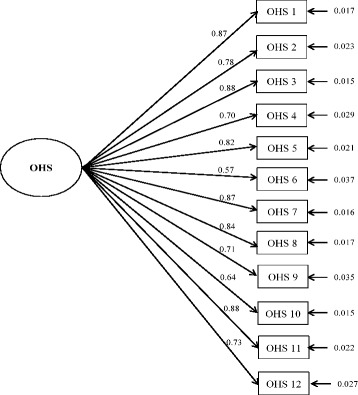
Confirmatory factor analysis for categorical data of the Oxford Hip Score (OHS) questionnaire. Standardized parameters and standard errors are shown. Fit indices are as follows: *χ* ^2^ = 174.6, degrees of freedom = 52, *p* <0.0001; RMSEA = 0.082 (CI 90%: 0.069 – 0.096); CFI = 0.982; TLI = 0.977

Hip OA greatly impacts the patient’s perception of health-related quality of life (HRQL) [6], and entails a great burden for the individual and the society as a whole. Studies of international scope have estimated that OA of the knee and hip constitute 0.7% of all disability adjusted life years (DALY) [7]. The DALYs lost due to hip OA increased 60% between 1990 and 2010 [8]. In the USA, the yearly expenditure resulting directly from hip OA was calculated to be $2827 per patient over 65 years of age (in the 1990s), and indirect costs can exceed that figure [1]. In Spain, the health-related expenses derived from OA can amount to 0.25 − 0.50% of the country GDP [9]. A study performed in Spain in 2007 estimated a yearly expenditure of €1500 per patient with hip or knee OA, 86% of which were direct costs [10].

It is necessary to incorporate the patient’s self-perception of health condition to the study of chronic diseases such as OA, both for appraising their current condition and the results of interventions [11]. The HRQL is a measure of the patient’s perception of their health condition that can be assessed via “generic” or “specific” tools. Generic tools are used to appraise health condition for any typology of patients, whereas specific tools are devised for a specific disease (e.g., OA of the hip), population segment (young vs. old), or type of problem (pain, dyspnea, *et cetera*) [12].

In the case of hip OA, there are several specific tools to evaluate HRQL, such as the Harris Score [13], the Western Ontario and McMaster Universities Osteoarthritis Index (WOMAC) for the assessment of OA of lower limbs [14], the Hip disability and Osteoarthritis Outcome Score (HOOS) for patients undergoing conservative treatment or surgery, the Hip Outcome Score [15] for patients about to undergo arthroscopy, or the Oxford Hip Score (OHS). Of them, only the WOMAC [16] and the Hip Outcome Score are validated in Spanish [17].

Although the OHS has been adapted to Spanish, its psychometric properties have not been assessed in the Spanish population setting. The OHS was designed to appraise the impact of total hip replacement surgery and was found to be more accurate than other generic questionnaires for that purpose [18]. Owing to its good psychometric properties, it has been favorably compared to other widely used tools that are more difficult to administer [19]. It has been adapted to Dutch [20], French [21], German [22], Italian [23], Danish [24], Turkish [25], and several Asian languages [26–28]. The questionnaire was also adapted to Spanish in Colombia and partially validated, although neither its sensitivity to changes nor factorial structure were checked [29]. Of all the mentioned adaptations, the factorial structure has only been validated by means of a confirmatory factor analysis in the original version of the OHS.

This study aims to assess the psychometric properties of the Spanish-adapted version of the OHS, including its factorial structure and other aspects of reliability, validity, and capacity to detect changes.

## Methods

### Design

Prospective, observational study, with a follow-up period for the recruited subjects of 6 months.

### Sampling and sample size

Opportunistic sampling was performed. Patients diagnosed with hip OA according to the criterion of the American College of Rheumatology [30] were recruited from traumatology, rheumatology, and primary care consultations in Vizcaya, Madrid, and Tenerife. Subjects were included in a consecutive way between January and December 2015. Not understanding Spanish, illiteracy, or being diagnosed with cognitive impairment were considered to be exclusion criteria.

Sample size was calculated on the basis of the most stringent analysis method employed: three hundred patients were estimated necessary to perform a confirmatory factorial analysis (CFA) using a single questionnaire that comprised 12 items [31]. This sample size allowed for estimating intraclass correlation coefficients (ICC) >0.8 with precision values ≥10% [32].

### Variables

The following personal characteristics were recorded for all participants: age, gender, body mass index (BMI), arthritis-affected joints, previous joint replacement surgery, and comorbidity, measured using the Charlson’s index [33]. In order to evaluate self-perception of HRQL, patients completed 3 questionnaires in their Spanish-adapted version: a generic one, the EQ-5D with a 5-level scale (EQ-5D-5 L) [34], and two specific ones, the WOMAC [14] and the OHS [18].

The EQ-5D-5 L [34] inquires about current self-perceived health condition and comprises two parts. The first part includes 5 questions on mobility, self-care, performance of daily-life activities, pain/discomfort, and anxiety/depression; each dimension is measured on a scale from 1 to 5; a single weighted score, called the utility index, is then obtained from these 5 questions, so that the greater the score the better the health condition [35]. The second part consists of a visual analogue scale (VAS) ranging from 0 (worst health condition) to 100 (best imaginable health condition).

The WOMAC [14] is a self-completed questionnaire, specifically aimed at patients suffering from OA of the hip or knee. Its multidimensional scale comprises 24 items clustered according to 3 dimensions: pain (5 items), stiffness (2 items), and physical functionality (17 items). A Likert-type scale was used with 5 possible answers to account for the intensity of each item (none, slight, moderate, severe, extreme), so each item receives a score from 0 to 4. The scores are then summed and standardized from 0 to 100 (best to worst), so that the greater the score the worse the patient’s health condition. This questionnaire has been adapted and validated for the Spanish setting [16].

The OHS is a self-administered questionnaire that can be completed via a personal interview or mailed by the patient after completion. It comprises 12 questions, with 5 possible answers each, to assess the patient’s perception of quality of life during the last 4 weeks. It has been employed with patients suffering from hip OA, both to study their baseline condition and to evaluate changes following prosthetic implant. Each answer receives a score from 0 to 4, where 4 is the best possible outcome [36]. A total score is obtained from the sum of all answers, ranging from 0 to 48, where 48 is the best possible outcome. The Spanish-adapted version was developed by performing a translation and linguistic validation using protocols consistent with internationally recognized good-practice guidelines under agreement with Oxford University Innovation ™ (see Additional file 1).

The recruited participants from Madrid were interviewed 7 to 14 days after the inclusion visit and, after verifying that there were no changes in their health condition, the OHS was repeated to check test-retest reliability.

All included patients were interviewed after a 6 months follow-up period: they were asked if they had undergone replacement surgery, all the questionnaires were repeated (EQ-5D-5 L, WOMAC, and OHS), and transition questions were posed to check for changes in their perception of global health.

### Statistical analysis

Continuous variables are described by their central tendency and dispersion, whereas qualitative variables are expressed by their percentages. Confidence intervals were set at 95% (CI 95%).

### Acceptability and ceiling and floor effects

The number of non-completed questionnaires and unanswered questions was recorded.

Ceiling or floor effects were considered to be present if more than 15% of respondents reported the highest or lowest possible score, respectively [37].

### Analysis of psychometric properties

#### Validity

The construct validity was assessed via an exploratory factor analysis (EFA). Barlett’s test of sphericity and a Kaiser-Meyer-Olkin (KMO) test were performed to evaluate the adequacy of employing such analysis. The null hypothesis of Barlett’s test states that the matrix of observed correlations is a singular matrix. Rejecting the null hypothesis allows for confirming the existence of linear relationships between factors and the explanatory variable. The KMO sampling adequacy test provides a measure of the variance among variables, and values >0.90 are considered optimal [38]. Factor loadings were calculated, with values >0.40 considered to be optimal, and so were communalities, that express the percentage of the item’s variance explained by each of the studied factors.

In order to complement our results and confirm the hypothesis of unidimensionality of the questionnaire, a confirmatory factor analysis (CFA) for categorical variables was carried out. The robust unweighted least squares estimator was used and the following fit indices were calculated [39, 40]: the root mean square error of approximation (RMSEA), for which a value <0.08 was acceptable, and the Tucker-Lewis Index (TLI) and Comparative Fit Index (CFI), both of which had to be >0.95 to be considered satisfactory [41]. Additionally, factor loadings were examined and those ≥0.40 were considered acceptable. The model was considered satisfactory if it surpassed these acceptability criteria.

The validity of the known groups was appraised by comparing the scores obtained in the OHS with each tercile of the EQ-5D-5 L and WOMAC distributions.

Convergent validity was assessed by calculating Pearson’s r or Spearman’s rho, which were then used to find the correlations between the scales of the OHS and those of the WOMAC and the EQ-5D-5 L. A threshold of 0.7 was set [37] for associations to be considered strong.

### Reliability

Internal consistency was assessed by calculating Cronbach’s α [42] for the scores obtained at the inclusion visit. This coefficient accounts for internal correlations of all items in a scale, so the greater Cronbach’s α is (range 0.0 to 1.0), the greater the consistency of the scale and the greater the probability that the questionnaire underlies a single dimension. In the case of a unidimensional tool comprising 12 items, Cronbach’s α is required to be >0.85 for the internal consistency to be considered optimal [43].

The test-retest reliability was checked using the ICC for comparing the scores of the test with the retest in the sub-sample from Madrid. According to the suggested classification for different reliability measurements [44], ICC values >0.7 are considered to be acceptable and >0.9 optimal.

### Sensitivity to change

The OHS questionnaire was repeated 6 months after the inclusion visit in order to evaluate its capacity to detect changes in the evolution of the disease. Transition questions were posed that inquired about the change in the hip condition perceived by the patient relative to the 6 previous months. Five possible answers (much worse, slightly worse, equal, slightly better, much better) were given and recorded on a scale. These questions aimed at appraising the sensitivity to change of the OHS questionnaire. Transition questions for the WOMAC were answered on the same scale, but they were specific for each of its domains (pain, stiffness, and physical functionality). Correlations between score changes in HRQL questionnaires and transition questions were assessed by calculating Spearman’s rho.

Changes in the OHS and EQ-5D-5 L questionnaires were calculated by subtracting initial from final scores, so positive values indicated an improvement in general condition. This was the opposite for the WOMAC, where final scores were subtracted from initial ones, and therefore positive values also indicated improvement. For each group of patients, baseline scores were then compared to those obtained at the follow-up period of 6 months to check if significant changes had occurred according to the transition questions. For every observed change, the effect size (ES) was calculated as the ratio between the mean and standard deviation (SD). Changes were considered to be moderate for values >0.5, and large for >0.8 [45]. Obtained values of ES were then compared to those of the WOMAC and EQ-5D-5 L scales. Responsiveness parameters were also estimated separately for patients who had suffered a hip arthroplasty and those who did not.

Additionally, the minimal clinically important difference (MCID) and the minimal detectable change (MDC) were estimated. These two measures correlate with responsiveness, but are more clinically oriented and focused at the individual level. The MCID was calculated using the mean change of patients that reported moderate improvement (feeling “slightly better”) at 6 months of the follow-up [46].

The MDC expresses the minimal magnitude of change above which the observed change is likely to be real and not just measurement error. The standard error of measurement (SEM), which represents the amount of error associated with a particular individual’s assessment, was estimated as the square root of the mean square error term from the ANOVA [47, 48]. From the SEM, the MDC was derived as follows [37, 47].

$$ \mathrm{MDC}=\mathrm{SEM} \times \mathrm{z}-\mathrm{score} \times \sqrt{2} $$

A 95% confidence level (MDC_95%_) was established, corresponding to a z-value of 1.96. The interpretation of MDC_95%_ is that if a patient has a change score equal to or higher than the MDC_95%_ threshold, it is possible to state with 95% confidence that this change is reliable and not the result of measurement error. Finally, to determine if the MCID surpassed the MDC_95%_, MCID was divided by the MDC_95%_ [49] so that if this ratio exceeded 1, the MCID could be discriminated from measurement error.

All effects were considered statistically significant at *p* < 0.05.

## Results

The study included 361 subjects: 157 from Vizcaya, 124 from Madrid, and 80 from Tenerife. Patients were recruited at primary care (37.7%), traumatology (46.5%), and rheumatology (10.8%) consultations. Women comprised 53.2% (CI 95%: 48.0–58.4%) of the sample and the average age was 67.8 years (CI 95%: 66.7–69.1 years).

Replacement of the contralateral hip had occurred in 17.5% (CI 95%: 13.6–21.4%) of cases. Charlson’s index had an average value of 0.8 points (CI 95%: 0.7–1.0), and mean BMI was 28.2 (CI 95%: 27.7–28.6).

Table 1 shows the outcome expressed by patients for the OHS, WOMAC, and EQ-5D-5 L questionnaires.

**Table 1:** Scores from the OHS, WOMAC, and EQ-5D-5 L questionnaires

	n	Mean score (CI 95%)	Median (Interquartile range)
OHS	359	22.8 (21.7–23.9)	22.0 (15.0–30.0)
WOMAC pain	360	45.8 (43.4–48.2)	45.0 (30.0–60.0)
WOMAC stiffness	360	48.3 (45.6–51.0)	50.0 (25.0–62.5)
WOMAC physical functionality	360	52.4 (50.0–54.8)	51.5 (36.8–70.6)
EQ-5D-5 L utility	357	0.52 (0.49–0.55)	0.61 (0.32–0.73)
EQ-5D-5 L VAS	357	54.5 (52.2–56.8)	50.0 (40.0–70.0)

Utility index score 0 to 1, where 0 = condition comparable to death, and 1 = perfect health condition. Negative scores are allowed

*OHS* Oxford Hip Score. Score range 1 to 48, the higher the score the better, *WOMAC* Western Ontario and McMaster Universities Osteoarthritis Index. Scale range 1 to 100, the higher the score the worse the condition

*VAS* Visual Analogue Scale. Score range 1 to 100, where 0 = worst possible health condition, and 100 = best imaginable health condition

### Acceptability and ceiling and floor effects

The obtained data allowed for summarizing the outcome of the OHS questionnaire in 359 cases (99.4%; CI95%: 98.7–100%). Questions 3 and 4 were answered in all cases, and questions 2, 5, 7, 8, and 10 in all but one. Questions 2, 6, and 11 were not answered in 2 occasions, and questions 9 and 12 in 3 occasions. The possible responses were the same for all questions, and so was the possible score range (0 to 4). There was no question for which more than 35% of the responses were concentrated at the top or lowest end of the scale: question 6 obtained the greatest percentage of responses for the lowest score (27.7%), and question 9 for the highest possible score (33.8%). For the total score, there was no aggregation at the low end of the scale and only 0.84% of the responses scored 48 out of 48 possible points in the inclusion visit. This score was reached by 3.08% of the patients that underwent hip replacement in the visit after six months. Hence, the presence of floor or ceiling effects was ruled out.

### Validity

In order to study the validity of the construct, an EFA was performed and a unidimensional structure was revealed with a single factor that explained 55.5% of variance (KMO = 0.945, Bartlett’s test of sphericity *χ* ^2^ = 2667, 66 degrees of freedom, *p* <0.001). Every factor loading was >0.50, and communalities were ≥0.40 with the exception of questions 6 and 10 (Table 2).

**Table 2:** Exploratory factor analysis of the Oxford Hip Score (OHS) items

	Exploratory factor analysis 1 factor	
Question: During the past 4 weeks…	Factor loading	Communality
During the past 4 weeks… How would you describe the pain you usually have from your hip? (OHS 1)	0.814	0.662
Have you had any trouble with washing and drying yourself (all over) because of your hip? (OHS 2)	0.791	0.626
Have you had any trouble getting in and out of a car or using public transport because of your hip? (OHS 3)	0.842	0.709
Have you been able to put on a pair of socks, stockings or tights? (OHS 4)	0.738	0.545
Could you do the household shopping on your own? (OHS 5)	0.797	0.635
For how long have you been able to walk before pain from your hip becomes severe? (OHS 6)	0.558	0.311
Have you been able to climb a flight of stairs? (OHS 7)	0.834	0.696
After a meal (sat at a table), how painful has it been for you to stand up from a chair because of your hip? (OHS 8)	0.810	0.656
Have you been limping when walking, because of your hip? (OHS 9)	0.698	0.488
Have you had any sudden, severe pain - ‘shooting’, ‘stabbing’ or ‘spasms’ - from the affected hip? (OHS 10)	0.630	0.396
How much has pain from your hip interfered with your usual work (including housework)? (OHS 11)	0.852	0.726
Have you been troubled by pain from your hip in bed at night? (OHS 12)	0.718	0.515

*OHS* Oxford Hip Score

Fit indices resulting from the performed CFA were adequate (Fig. 1): (a) the RMSEA was 0.082, a very close value to the set threshold of 0.08; and (b) the CFI and TLI were 0.982 and 0.977, respectively, both exceeding the benchmark of 0.95. All factor loadings were statistically significant (*p* <0.001), ranging from 0.57 to 0.88.

Validity of known groups, which is a measure of the questionnaire’s discriminatory capacity, can be observed in Table 3. It shows average scores of the OHS with their CI95% for each tercile of the distributions of the WOMAC and EQ-5D-5 L scales. Differences between the 3 groups are clearly shown by the OHS scores, with average changes of 4.7 to 12.0 points per tercile.

**Table 3:** Average scores of the OHS. Patients are classified according to the terciles obtained from the WOMAC and EQ-5D-5 L questionnaires

	OHS score for the lower tercile of the distribution (CI 95%)	OHS score for the middle tercile of the distribution (CI 95%)	OHS score for the top tercile of the distribution (CI 95%)
WOMAC pain	32.2 (30.7–33.6)	22.7 (21.1–24.2)	13.7 (12.6–14.9)
WOMAC stiffness	29.7 (28.1–31.2)	22.1 (20.8–23.4)	14.4 (13.0–15.7)
WOMAC physical functionality	33.6 (32.3–34.9)	21.6 (20.6–22.6)	13.0 (11.9–14.1)
EQ-5D-5 L utility	12.7 (11.7–13.7)	22.4 (21.2–23.5)	32.4 (31.1–33.9)
EQ-5D-5 L VAS	15.5 (14.0–17.0)	24.0 (22.3–25.7)	28.7 (26.7–30.5)

*OHS* Oxford Hip Score, *WOMAC* Western Ontario and McMaster Universities Osteoarthritis Index, *VAS* Visual Analogue Scale

Table 4 shows the correlations between the OHS scores and the WOMAC domains or the EQ-5D-5 L domains, utility index and VAS. Given the different types of scale measures, negative correlations with the WOMAC and positive ones with the EQ-5D-5 L were to be expected. All associations were strong except for the stiffness scale of the WOMAC questionnaire, where the correlation was at the limit of the set threshold, and the EQ-5D-5 L VAS.

**Table 4:** Correlations between the OHS score and the WOMAC scales or the EQ-5D-5 L

	OHS	
	*r/rho*	*p*-value
WOMAC		
Pain	−0.807^a^	<0.001
Stiffness	−0.686^a^	<0.001
Functionality	−0.893^a^	<0.001
EQ-5D-5 L		
Mobility,	−0.768^b^	<0.001
Self-care,	−0.728^b^	<0.001
Performance of daily-life activities	−0.748^b^	<0.001
Pain/discomfort	−0.778^b^	<0.001
Anxiety/depression	−0.562^b^	<0.001
Utility Index	0.835^a^	<0.001
VAS	0.575^a^	<0.001

Higher scores in the OHS and EQ-5D-5 L indicate better health condition, and the opposite happens in the case of the WOMAC

*OHS* Oxford Hip Score, *WOMAC* Western Ontario and McMaster Universities Osteoarthritis Index

^a^ Pearson’s r

^b^ Spearman’s rho

Correlations between the scores of the WOMAC scales on pain, stiffness, and physical functionality and the EQ-5D utilities were −0.769, −0.628, and −0.829, respectively (all values were statistically significant, *p* <0.001). Correlations between the scores of the WOMAC scales on pain, stiffness, and physical functionality and the EQ-5D VAS were −0.563, −0.410, and −0.560, respectively (all values were statistically significant, *p* <0.001).

### Reliability

Internal consistency was assessed via Cronbach’s α, which was calculated to be 0.928 for the OHS questionnaire. For the 124 subjects that repeated the questionnaire 7 to 14 days after their inclusion in the study, ICC was 0.992 (CI 95%: 0.994–0.998).

### Sensitivity to change

A follow-up on 313 subjects took place after 6 months. Of them, 65 had undergone hip replacement surgery and 94 (30.0%, CI 95%: 25.0–35.1%) reported feeling “slightly better” or “much better” on the side of the hip for which they entered the study. Of the follow-up sample, 133 (42.5%; CI 95%: 37.0–48.0%) stated feeling “slightly worse” or “much worse”.

Table 5 shows the mean change in the scores obtained from the employed questionnaires when the patient had perceived a change in their health condition. First, the correlations between score changes between the HRQL questionnaires and transition questions were assessed. The correlation between the change in the OHS score and transition questions was moderate (Spearman’s rho = 0.636, *p* <0.0001). The correlations between changes in the domains of the WOMAC and specific transition questions were also moderate (Spearman’s rho absolute value between 0.544 and 0.635; *p* <0.0001)

**Table 5:** Changes observed in the OHS, EQ-5D-5 L, and WOMAC questionnaires at 6 months of the follow-up period, in patients that reported health condition changes

	The hip condition is “much worse” *N* = 53		The hip condition is “slightly worse” *N* = 78		The hip condition is “slightly better” *N* = 34		The hip condition is “much better” *N* = 60	
	Average change (CI 95%)	E.S.	Average change (CI 95%)	E.S.	Average change (CI 95%)	E.S.	Average change (CI 95%)	E.S.
OHS	−5.8 (−8.1–−3.4)	0.69	−2.4 (−3.6–−1.1)	0.42	7.0 (3.7–10.4)	0.73	18.7 (15.9–21.6)	1.71
EQ-5D-5 L utility	−0.16 (−0.23–−0.08)	0.57	−0.06 (−0.11–−0.01)	0.09	0.19 (0.10–0.28)	0.73	0.40 (0.33–0.47)	1.48
EQ-5D-5 L VAS	−5.3 (−12.9–2.2)	0.20	−1.6 (−5.6–2.4)	0.09	9.0 (0.6–17.3)	0.37	20.6 (14.7–26.5)	0.91
	Pain is “much worse” *N* = 46		Pain is “slightly worse” *N* = 77		Pain is “slightly better” *N* = 42		Pain is “much better” *N* = 60	
	Average change (CI 95%)	E.S.	Average change (CI 95%)	E.S.	Average change (CI 95%)	E.S.	Average change (CI 95%)	E.S.
WOMAC pain	−13.5 (−18.5–−8.4)	0.79	−4.1 (−7.2–−1.0)	0.30	14.9 (8.5–21.3)	0.73	38.3 (32.2–44.3)	1.64
	Stiffness is “much worse” *N* = 38		Stiffness is “slightly worse” *N* = 82		Stiffness is “slightly better” *N* = 33		Stiffness is “much better” *N* = 56	
	Average change (CI 95%)	E.S.	Average change (CI 95%)	E.S.	Average change (CI 95%)	E.S.	Average change (CI 95%)	E.S.
WOMAC Stiffness	−12.2 (−20.9–−3.4)	0.50	−6.9 (−11.5–6.2)	0.32	17.8 (8.5–27.1)	0.68	38.6 (31.1–46.1)	1.37
	Physical functionality is “much worse” *N* = 48		Physical functionality is “slightly worse” *N* = 82		Physical functionality is “slightly better” *N* = 33		Physical functionality is “much better” *N* = 47	
	Average change (CI 95%)	E.S.	Average change (CI 95%)	E.S.	Average change (CI 95%)	E.S.	Average change (CI 95%)	E.S.
WOMAC Physical functionality	−8.7 (−14.5–−2.9)	0.43	−4.9 (−8.4–−1.5)	0.31	21.3 (14.0–28.7)	1.03	43.2 (36.2–50.2)	1.81

Final scores were subtracted from basal scores in the OHS and EQ-5D-5 L, and the opposite happened in the WOMAC. This way, positive outcomes always indicate improvement

*E.S*. Effect Size, *OHS* Oxford Hip Score, *WOMAC* Western Ontario and McMaster Universities Osteoarthritis Index

The ES of the change in the OHS was 0.73 for subjects that reported feeling “slightly better” and 1.71 for those that reported feeling “much better”. Sensitivity to change obtained lower ES values for negative changes, with values of 0.42 and 0.69 in the case of subjects reporting “slightly worse” and “much worse”, respectively. A clear gradient in the scores was observed that depended on the change perceived by the patient, which was significantly different for those feeling “slightly worse”, “slightly better”, and “much better”. There was a small overlap between subjects feeling “much worse” and “slightly worse”. The OHS questionnaire proved to be a more sensitive tool than the EQ-5D-5 L, and similar to the WOMAC.

Table 6 shows the mean change in the scores obtained from the OHS questionnaires for both patients that had undergone hip arthroplasty and those who did not. Results were consistent with those from the whole sample, although improvements perceived by patients who underwent hip arthroplasty were significantly greater.

**Table 6:** Changes observed in the OHS questionnaires at a 6 months follow-up for patients having undergone hip arthroplasty or not

Not Arthroplasty	The hip condition is “much worse” *N* = 51		The hip condition is “slightly worse” *N* = 76		The hip condition is “slightly better” *N* = 19		The hip condition is “much better” *N* = 14	
	Average change (CI 95%)	E.S.	Average change (CI 95%)	E.S.	Average change (CI 95%)	E.S.	Average change (CI 95%)	E.S.
OHS	−5.8 (−8.1–−3.4)	0.69	−2.4 (−3.6–−1.1)	0.42	3.6 (−0.9–8.0)	0.39	12.4 (6.8–17.9)	1.28
Arthroplasty	The hip condition is “much worse” *N* = 1		The hip condition is “slightly worse” *N* = 2		The hip condition is “slightly better” *N* = 15		The hip condition is “much better” *N* = 46	
OHS	−2.00	–	−3.5 (−60.7–53.7)	0.55	11.4 (6.7–11.1)	1.35	20.7 (17.5–23.8)	1.94

Final scores were subtracted from basal scores

*E.S*. Effect Size, *OHS* Oxford Hip Score

The average change in the OHS scores was 7.0 points (SD = 9.6) in the case of subjects that felt moderate improvement, which was the figure used for calculating the MCID. The SEM was calculated to be 2.0 and hence, the estimated value of MDC_95%_ was 5.5. The obtained ratio MCID/MDC_95%_ was 1.3.

## Discussion

The Spanish version of the OHS is a valid tool for measuring HRQL in patients suffering from hip OA, and is both reliable and sensitive to changes. Additionally, it is very well accepted by the population it addresses, as proven by the extraordinarily high response rate, although in this case it could be influenced by the way in which it was administered, in the clinical setting.

The validity of the OHS has been assessed from different perspectives, although apparent validity was not one of them given it is an adaptation.

The validity of known groups, namely its discriminatory validity, appears to be adequate since scores differ between subjects classified according to their HRQL, via specific or general questionnaires. Significant ceiling or floor effects that could compromise such discriminatory capacity were not found. The presence of floor effect has not ever reported with the OHS. On the contrary, certain studies have observed a ceiling effect in postoperative patients [24], although the majority have not [21, 22, 50]. The previously shown results rule out the presence of ceiling effect, even in patients who had undergone hip replacement, in agreement with the results of the original version [51].

The analysis of convergent validity showed correlations with the specific scales of the WOMAC and the generic scales of the EQ-5D-5 L. Such correlations were stronger than those found between the original questionnaire and generic tools for measuring HRQL [51], and similar or slightly stronger than adapted versions of the OHS to other languages, such as German [22] or Dutch [20].

The construct validity was also part of the validation. The factorial structure of the OHS has been previously discussed, and several authors have proposed to differentiate 2 domains within it: pain and physical functionality [52]. When attempting to check if a single- or double-factor structure worked better, the outcome supported both possibilities, although there were several items that saturated both factors when considering a bidimensional structure [53]. For these reasons and in view of the outcome of the performed EFA, which was similar to other adapted versions [54], a unidimensional structure was tested, which seemed a correct approach for this adaptation given that the values obtained in the CFA were close to the acceptability threshold for the RMSEA and optimal for the TLI and CFI [41].

Cronbach’s α, which accounts for internal consistency, was better than for the original scale at the inclusion visit (0.93 vs 0.84) [55]. Although a very high value of Cronbach’s α could indicate that the items are redundant, this is unlikely to be the case, since it was ruled out by the factorial analysis. This coefficient is useful for estimating reliability, particularly for a unidimensional test. If a test shows a high value of α, then it can be concluded that its variance is largely attributable to general and group factors. When the existence of a single factor has been demonstrated, then Cronbach’s α can be used to conclude that the set of items is unidimensional [43].

Test-retest reliability was measured via ICCs and found to be excellent, with values >0.90 that in a sample of 124 patients allows for classifying the tool as reliable [37]. Reported values of ICC were slightly higher than those found in other studies (range from 0.89 to 0.97) [22–24, 27, 50], which may be due to the way in which the OHS score was obtained in the follow-up, namely by telephone interview.

The reliability study of this adapted version of the OHS yielded values of internal consistency and reliability that were similar to another Spanish-validated tool, the Hip Outcomes Score, which is also designed to appraise changes in perceived HRQL by patients following hip surgery [17].

The discriminatory capacity of the questionnaire, which accounts for its potential to discriminate patients in different situations, was satisfactory; however, the tool has also proven its usefulness to study the subject’s perception of change in their own situation, that is, its evaluative capacity is adequate [11]. The instrument was originally designed for this purpose and this study has confirmed the potential of its adaptation to Spanish, not only in patients that undergo hip surgery but also in the short-term evolution of a cohort of patients suffering from hip OA.

The ES for “moderate” positive changes showed values that were slightly under the set threshold of 0.8 points, and similar to those of the WOMAC. The ES for changes following surgery was 1.93 in the validation process of the original questionnaire [56], which is only comparable with improvements in patients that underwent hip replacement (ES = 1.35). The OHS proved to be superior to other questionnaires, like the WOMAC and the generic EQ-5D-5 L, when assessing significant changes, as is the case of hip replacement [51]. In this work, the OHS showed a similar capacity for detecting “moderate” changes compared to the WOMAC, but was slightly better when examining “significant” changes.

For subjects that reported feeling a “moderate” improvement in their condition, MCID was 7.0 points. For the original version of the OHS, MCID was calculated from the scores reported by patients that had underdone hip replacement surgery, and values of 7.5 points were obtained [57]. Using the criterion of estimating MCID as half the SD of the distribution of scores given by subjects that had experienced changes [58], MCID can be calculated to be around 4.8 points. According to this criterion, the values of MCID for the original version would be between 3 and 5 points, similarly to those obtained in our study [36].

In agreement with other studies, the evaluative capacity was greater for detecting positive rather than negative changes [59], although the capacity observed for the OHS to detect negative changes was similar or greater than the WOMAC and, of course, than that of generic questionnaires like the EQ-5D-5 L.

The MDC_95%_ was 5.5 points as calculated from the SEM, a value that is similar to the original questionnaire (MDC_90%_ = 4.85 points) [57]. The MDC represents the lowest score change (at the particular patient level) that is not the result of measurement error of the instrument. The MDC is based on the standard error of measurement, which depends on the accuracy and variability of its components [47], and can be understood as the lowest bound of real change, although it may not indicate clinical significance. The ratio between the MCID and MDC_95%_ was higher than 1, indicating that the MCID can be discriminated clearly from measurement error.

There are some limitations to this work. The studied sample may not be representative of the Spanish population, despite including patients from different geographic regions and at various stages of the disease evolution. On the other hand, the used methodology (the classical test theory), with its assumptions and constraints, entails certain limitations to evaluate psychometric properties; in order to overcome them, the validation process has been complemented by performing a CFA specific for categorical data, which employs statistical analysis to validate *a priori* made assumptions [39].

Traditionally, the OHS has been used to assess the impact of hip replacement surgery [60] on HRQL, as well as other surgical [50] or non-surgical [23] procedures. Few studies have focused on its discriminatory capacity [25, 29]. This work highlights the discriminatory capacity of the tool and appraises its sensitivity to changes in the general evolution of the disease, including both patients that undergo joint replacement and those who do not. Its usefulness is similar to other instruments having a broad experience of use after being adapted to other languages, like the WOMAC, and displays greater capability to detect changes than generic tools like the EQ-5D-5 L.

## Conclusions

The Spanish adaptation of the OHS is a useful instrument to assess perception of HRQL in patients suffering from hip OA, being well-accepted, and with good psychometric properties that support its use for evaluating a patient’s condition at a given moment, and for appraising changes over time.

Incorporating this kind of tools to usual clinical practice will facilitate the valid and reliable evaluation of a patient’s self-perceived health condition and the outcome of interventions, both at the individual and population level.

## Additional file

Additional file 1: Adapted version of the Oxford Hip Score - Spanish (Spain). (DOC 47 kb)

## Abbreviations

BMI: Body mass index
CFA: Confirmatory factor analysis
CFI: Comparative fit index
CI95%: 95% confidence interval
EFA: Exploratory factor analysis
ES: Effect size
GDP: Gross domestic product
HRQL: Health-related quality of life
ICC: Intraclass correlation coefficient
KMO: Kaiser-Meyer-Olkin
MCID: Minimal clinically important difference
MDC: Minimal detectable change
OA: Osteoarthritis
OHS: Oxford hip score
RMSEA: Root mean square error of approximation
SEM: Standard error of measurement
TLI: Tucker-lewis index
VAS: Visual analogue scale
WOMAC: Western Ontario and McMaster Universities Osteoarthritis Index
