# Supplementary material for: Cross-cultural adaptation and validation of the Spanish version of the Oxford Hip Score in patients with hip osteoarthritis
Source: BMC Musculoskelet Disord. 2017 May 22;18:205. doi: 10.1186/s12891-017-1568-3 (PMC5440982; doi:10.1186/s12891-017-1568-3)
Supplement: Additional file 1: — Adapted version of the Oxford Hip Score - Spanish (Spain). (DOC 47 kb) [file 12891_2017_1568_MOESM1_ESM.doc]

**Adapted version of the Oxford Hip Score - Spanish (Spain).**

**© Oxford University Innovation Limited, 1998. All rights reserved.**

1. Durante las últimas 4 semanas, ¿cómo describiría el dolor que tiene normalmente debido a la cadera?

| Ninguno | Muy leve | Leve | Moderado | Grave |
| --- | --- | --- | --- | --- |
| ⬜ | ⬜ | ⬜ | ⬜ | ⬜ |

1. Durante las últimas 4 semanas ¿ha tenido algún problema para lavarse y secarse (todo el cuerpo) debido a su cadera?

| Ningún problema | Muy pocos problemas | Problemas moderados | Dificultad extrema | Imposible hacerlo |
| --- | --- | --- | --- | --- |
| ⬜ | ⬜ | ⬜ | ⬜ | ⬜ |

1. Durante las últimas 4 semanas, ¿ha tenido algún problema para subir y bajar de un coche o para usar el transporte público debido a su cadera? (sea cual sea el que suela utilizar)

| Ningún problema | Muy pocos problemas | Problemas moderados | Dificultad extrema | Imposible hacerlo |
| --- | --- | --- | --- | --- |
| ⬜ | ⬜ | ⬜ | ⬜ | ⬜ |

1. Durante las últimas 4 semanas, ¿se ha podido poner un par de calcetines, medias o pantis?

| Sí, fácilmente | Con poca dificultad | Con dificultad moderada | Con extrema dificultad | No,  imposible |
| --- | --- | --- | --- | --- |
| ⬜ | ⬜ | ⬜ | ⬜ | ⬜ |

1. Durante las últimas 4 semanas, ¿podría hacer la compra para su casa usted solo/a?

| Sí, fácilmente | Con poca dificultad | Con dificultad moderada | Con extrema dificultad | No, imposible |
| --- | --- | --- | --- | --- |
| ⬜ | ⬜ | ⬜ | ⬜ | ⬜ |

1. Durante las últimas 4 semanas, ¿durante cuánto tiempo ha podido andar antes de que el dolor debido a su cadera se volviera grave (con o sin bastón)?

| Sin dolor/Más de 30 minutos | 16 a 30 minutos | 5 a 15 minutos | Solo por casa | Nada/dolor grave al caminar |
| --- | --- | --- | --- | --- |
| ⬜ | ⬜ | ⬜ | ⬜ | ⬜ |

1. Durante las últimas 4 semanas, ¿ha podido subir un tramo de escaleras?

| Sí, fácilmente | Con poca dificultad | Con dificultad moderada | Con extrema dificultad | No, imposible |
| --- | --- | --- | --- | --- |
| ⬜ | ⬜ | ⬜ | ⬜ | ⬜ |

1. Durante las últimas 4 semanas, tras una comida (sentado/a a una mesa), ¿en qué medida le ha resultado doloroso levantarse de la silla debido a su cadera?

| Nada doloroso | Ligeramente doloroso | Moderadamente doloroso | Muy  doloroso | Insoportable |
| --- | --- | --- | --- | --- |
| ⬜ | ⬜ | ⬜ | ⬜ | ⬜ |

1. Durante las últimas 4 semanas, ¿ha estado cojeando al andar debido a su cadera?

| Rara vez/ nunca | A veces, o solo al principio | Con frecuencia, no solo al principio | La mayoría del tiempo | Todo el tiempo |
| --- | --- | --- | --- | --- |
| ⬜ | ⬜ | ⬜ | ⬜ | ⬜ |

1. Durante las últimas 4 semanas, ¿ha tenido algún dolor grave y repentino: “fulgurante”, “punzante” o “espasmos”, debido a la cadera afectada?

| Ningún  día | Solo 1 o 2  días | Algunos  días | La mayoría de días | Todos los  días |
| --- | --- | --- | --- | --- |
| ⬜ | ⬜ | ⬜ | ⬜ | ⬜ |

1. Durante las últimas 4 semanas, ¿en qué medida ha interferido su dolor debido a la cadera con su trabajo habitual (incluidas las tareas domésticas)?

| Nada | Un poco | Moderadamente | Mucho | Totalmente |
| --- | --- | --- | --- | --- |
| ⬜ | ⬜ | ⬜ | ⬜ | ⬜ |

1. Durante las últimas 4 semanas ¿le ha molestado el dolor debido a la cadera en la cama por la noche?

| Ninguna  noche | Solo 1 o 2 noches | Algunas  noches | La mayoría de noches | Todas las  noches |
| --- | --- | --- | --- | --- |
| ⬜ | ⬜ | ⬜ | ⬜ | ⬜ |

All licenses to use the Oxford Hip Score in Spanish should be requested from Oxford University Innovation Ltd using the following link https://innovation.ox.ac.uk/clinical-outcomes/patient-reported-outcome-measures/
